# An Evaluation of the Cost of Human Papilloma Virus (HPV) Vaccine Delivery In Zambia

**DOI:** 10.21203/rs.3.rs-2919637/v1

**Published:** 2023-05-29

**Authors:** Moses C Simuyemba, Chitalu M Chama-Chiliba, Abson Chompola, Aaaron Sinyangwe, Abdallah Bchir, Gilbert Asiimwe, Felix Masiye, Carla Chibwesha

**Affiliations:** University of Zambia School of Public Health; University of Zambia; University of Zambia; University of Zambia; University of Monastir; Gavi; University of Zambia; University of North Carolina at Chapel Hill

**Keywords:** HPV vaccine, Human Papilloma Virus, Zambia, Cost of HPV vaccination, Gavi, HPV vaccine in Africa

## Abstract

**Background:**

Human papillomavirus (HPV) is a common sexually transmitted infection and the leading cause of cervical cancer. The HPV vaccine is a safe and effective way to prevent HPV infection. In Zambia, the vaccine is given during Child Health to girls aged 14 years who are in and out of school in two doses over two years. The focus of this evaluation was to establish the cost to administer a single dose of the vaccine well as for full immunisation of two doses.

**Methods:**

For HPV costing, both top-down and micro-costing approaches were used, depending on the cost data source, and economic costs were gathered from Expanded Programme for Immunisation Costing and Financing Project (EPIC). Data was collected from eight districts in four provinces, mainly using a structured questionnaire, document reviews and key informant interviews with staff at national, district and provincial levels.

**Results:**

Findings show that schools made up 53.3% of vaccination sites, community outreach sites 30.9% and finally health facilities 15.8%. In terms of coverage for 2020, for the eight districts sampled, schools had the highest coverage at 96.0%. Community outreach sites were at 6.0% of the coverage and health facilities accounted for only 1.0% of the coverage. School based delivery had the lowest cost economic cost at USD13.2 per dose and USD 26.4 per fully immunised child (FIC). Overall financial costs were US$6.0 per dose and US$11.9 per fully immunised child. Overall economic costs taking all delivery models into account were US$23.0 per dose and US$46.0 per FIC. The main cost drivers were human resources, building overhead and vehicles, microplanning, supplies and service delivery/outreach. were the top cost drivers. Nurses, environmental health technicians and community-based volunteers were the most involved in HPV vaccination.

**Conclusions:**

Future planning in Zambia and other African countries conducting HPV vaccination needs to prioritise these cost drivers as well as possibly find strategies to minimise some costs. Although not a challenge now due to Gavi support, vaccine costs are a major threat to sustainability in the long run. Countries like Zambia must find strategies to mitigate against this.

## Background

Human papillomavirus (HPV) is a common sexually transmitted infection that can cause a range of health problems, including genital warts and certain types of cancer.(1–3) The HPV vaccine is a safe and effective way to prevent HPV infection and the health problems it can cause.(1, 2, 4)

Since 2019, the HPV vaccine in Zambia is provided through the country’s Expanded Programme on Immunisation (EPI), once a year during the Child Health Week (CHW) in the month of June, targeting girls aged 14 years who are in and out of school. The overall target for the programme is girls aged 9 to 14, but so far only the 14-year-olds have been vaccinated owing to inadequate doses of HPV vaccine being available on the global market and thus Gavi could only secure a limited number of vaccines.

The EPI decided to vaccinate 14-year-olds first as this was their last opportunity to receive the vaccine before they became ineligible age wise. HPV vaccine delivery in Zambia typically relies on a range of different delivery platforms, including health facilities, schools, and community-outreach sites. These different delivery platforms are used to ensure that the HPV vaccine is widely available and accessible to those who need it, and to increase vaccine uptake. The main focus in terms of social mobilisation and messaging has been to get as many girls vaccinated in schools and the rest, mainly those out of school, using health facilities and outreach sites. The Ministry of Health (MOH), which works with a range of partners, including international organizations and non-governmental organizations, to ensure that the vaccine is widely available and accessible to those who need it as well as to educate the public about the importance of vaccination and to dispel myths and misconceptions about the vaccine.

Gavi, the Vaccine Alliance is a global organization that works to increase access to vaccines in low- and middle-income countries. In Zambia, Gavi has played a vital role in supporting HPV vaccine delivery by providing funding to the country’s national immunization program to help cover the costs of purchasing and distributing the vaccine.(5) Gavi has also provided technical assistance and capacity building to help the Ministry of Health implement effective strategies for increasing vaccine uptake, and has supported the training of health care workers to administer the vaccine safely and effectively. Overall, Gavi’s support has helped to ensure that more girls and young women in Zambia have access to the HPV vaccine.(5)

In recommending HPV vaccine introduction the Zambia Immunisation Technical Advisory Group, ZITAG, used cost and cost-effectiveness studies from Brazil, Canada, the United Kingdom, and Tanzania.(6, 7) The transferability of such findings to the Zambian context was arguable and such decisions were often heavily influenced by global level directives made by donors (Wilkinson, Sculpher et al. 2016).(6)

A PATH study during the demonstration project estimated the financial cost at between US$9.98 and US$10.40 per fully immunised girl (PATH 2014) but it did not explore cost-effectiveness.(8) A Gavi Full Country Evaluation (FCE) report stated that the financial and programmatic sustainability implications of introducing HPV vaccine nationally were not thoroughly assessed using local evidence and recommended that the Ministry of Health develop clear policy and guidelines for purposes of economic evaluation of new vaccine introductions.(6) As Zambia proceeds with the national introduction the question is now centred around understanding the cost of vaccination for a nationwide programme compared to a demonstration project in only one district. This is the focus of this paper, to establish the cost to administer as single dose as well as to get a girl fully vaccinated across the three delivery platforms being utilised, now that HPV vaccination is nationwide. The paper also highlights an approach that can be taken to apportion costs across different delivery platforms in settings where such apportionment may be challenging.

## Methods

For HPV costing, both top-down and micro-costing approaches were used, depending on the source of the cost data, to estimate the financial and economic costs of implementing HPV using the delivery model of child health week.(9) Micro-costing is a method of estimating the costs of delivering a specific intervention or program at a detailed level and involves identifying and quantifying all of the resources and activities that are needed to deliver the intervention or program, and then assigning a cost to each of these elements. Top-down costing is a high-level approach that estimates the overall cost of a program or service based on aggregated data and in this case this was data from national level budgets of the Expanded Programme on Immunisation. Table 1 summarises the resource input categories. Some costs were gathered from 2019 and others from 2020 vaccination week and the 2019 costs were inflated to 2020 equivalent at 9.15% inflation rate.(10) Costs were gathered in both USD and ZMW and exchange rate of ZMW18.36 was used for 2020, based on Bank of Zambia exchange rate data.(11)

Data for these costs was collected at both national and subnational levels mainly using a structured questionnaire, supplemented by document reviews (HPV microplans, reports, budgets) and key informant interviews with staff at national, district and provincial levels. A sample of eight districts in four provinces was included in order to be more representative than the earlier demonstration phase costing. The sample for the subnational level built on the EPI Costing and Financing Project (EPIC) sample of 2011 with inclusion of an additional province in consultation with the Ministry of Health.(12) These were Lusaka province (Lusaka and Chongwe districts); Copperbelt province (Ndola and Lufwanyama districts); Central province (Kabwe and Mkushi districts) and Muchinga province as the addition (Mpika and Chinsali districts). Expenditure data on all key activities, including planning, social mobilization, vaccines and other supplies, service delivery, supervision, data collection and compilation, was included.

Data analysis varied according to data source and level of data. For national level data, costs were allocated to each district if they were budgeted as such. Costs that were not specific to a district were allocated by dividing the total cost by the number of districts in the country. For data collected from the eight sampled districts, total costs for all eight districts and average costs per district were calculated. The total costs were apportioned to each delivery model (school, health facility and outreach) based on the proportion of vaccination sites utilised for each model. This approach was used as it was generally not possible to allocate specific costs to each delivery model due to the manner in which budgets at district level were made and utilised without detailed allocation to each delivery model. Economic costs were gathered from EPIC study data. Univariate and multivariate sensitivity analysis was carried out to assess the output with cost per dose as the dependent variable and all cost inputs listed in Table 4 as independent variables, adjusting them between 1–10%.(13–15)

## Findings

Table 1 provides details of the three delivery models used in Zambia for HPV vaccine delivery across the eight sampled districts. Schools made up more than half the sites for vaccination, followed by community outreach sites and finally health facilities. This was used as a basis for apportioning costs later.

In terms of coverage for 2020, for the eight districts sampled, schools had the highest coverage, averaging 87.7% of the coverage across first and second doses. Community outreach sites were at 5.8% of the coverage and health facilities accounted for only 1.0% of the coverage as shown in Table 2.

Table 3 gives details of the costs attributable to HPV vaccination in Zambia. As described in the methodology, this is a combination of data from different sources, including national and district level data. For economic costs, EPIC data was utilised. Note that costs were gathered from 2019 and 2020 and average figures worked out or in the case where a cost was not available in 2020, due to funding limitations, 2019 figures were used.

As illustrated in Table 4, human resource-related costs remain the largest costs associated with the EPI programme and HPV vaccination is no exception. In the table above, most of the costs, other than vaccine costs, related to staff allowances, per diem and fuel (microplanning, training, service delivery and outreach). Enrolled Nurse/Midwife position accounted the largest proportion of time spent on HPV vaccination by all cadres at 27.4%. This was followed by Registered Nurse/Midwife at 24.7%, Environmental Health Technologist (EHT) at 12.2%, Community based volunteers (CBV) at 11.2% and clinical officers at 6.6%. Social mobilisation and supervision/monitoring and evaluation were highly underfunded, according to key informants who stated that it was inadequate.

Finally, Table 5 provides the calculated costs based on the foregoing tables. The total costs was apportioned to each delivery model based on the data in Table 1 on the proportion of delivery sites for each model. This was divided by the coverage (dose 1 and dose 2) for each model as outlined in Table 2 to arrive at ethe cost per dose. The cost per fully immunised child was arrived at by multiplying this average cost by 2 for two doses for fully immunised status. School based delivery had the lowest cost economic cost at USD13.2 per dose and 26.4 per fully immunised child. Financial costs were US$6.0 per dose and US$11.9 per fully immunised child. Overall economic costs taking all delivery models into account were US$23.0 per dose and US$46.0 per FIC.

Sensitivity analysis showed that health facility delivery is most affected by changes in costs, mainly due to the low coverage achieved there. Schools were least sensitive to changes in costs due to higher coverage.

## Discussion

The cost of administering HPV vaccine across different delivery platforms in Africa is a critical factor in determining the success of HPV vaccination programs.(15–19) School-based delivery has been shown to be the most cost-effective method for delivering the vaccine, as it requires minimal resources and can be implemented quickly with higher coverage in most countries.(16, 18, 20, 21) The findings of this evaluation support this as school based HPV vaccine delivery had the highest coverage and lowest cost per dose. According to key informants, orientation and social mobilisation efforts were highly focused on the schools and less so on health facility access and outreach. This may disadvantage out-of-school girls in terms of accessing the vaccine.(16) Additionally, personnel based at health facilities were not so focused on HPV vaccination, despite provision for this, although this and community outreach were meant to cater for that category of girls, a hard-to-reach population.(16) In Cambodia and Zimbabwe, out-of-school girls identified by community health workers were and invited to come to schools for vaccination, But the effectiveness of this approach could not be shown, illustrating the enormity of the challenge.(18, 22)

Health facility-based delivery is also an effective option, although the cost of facility-based delivery is higher due to the need for additional equipment and personnel and usually lower coverage in most countries that have implemented this approach.(16, 18) Community-based delivery is an important option for reaching rural and hard-to-reach populations, but the cost of community-based delivery is often higher than school-based or health facility-based delivery due to logistical challenges and the need for additional resources.(18) Cost-effectiveness studies have shown that the cost of delivering the HPV vaccine through different delivery platforms in Africa can vary significantly, depending on the type of delivery platform, the population being targeted, and the resources available.(16)

A study of costs of HPV vaccination n Gavi supported countries using the World Health Organization Cervical Cancer Prevention and Control Costing Tool found the average economic cost per dose to have been US$19.98 and US$8.74 as the financial cost across one year demonstration projects.(16) The economic cost of US$23.0 per dose for Zambia from this study is compared to other available studies in the African countries shown in Table 6.(17–19, 21, 23) The highest economic cost per dose at US$45.0 was in Zimbabwe and the lowest at US$3.09 was in Mwanza district in Tanzania, although the national average of US$10.62 for Tanzania is better comparison.

In terms of financial costs the main cost drivers in Zambia were supplies (e.g. syringes, needles, safety boxes etc) as well as costs associated with per diems and allowances for staff and community workers and volunteers (relating to microplanning, training, orientation, social mobilisation and monitoring and evaluation as well as actually vaccination activities). When we consider economic costs human resource costs were the largest, followed by buildings and vehicles and then cold chain equipment and maintenance.

Human resource related costs in terms of per diems, allowances and salaries were the largest cost drivers for most of the countries whose studies have been included here.(17–19, 21, 23) As shown in the results, nurses (both enrolled and registered) made up over half of the total time spent by all cadres on HPV immunisation activities. This is less surprising perhaps than being the next most utilised cadres followed by CBV. The EHTS took up roles related to social mobilisation and community outreach due to lack of specific cadres employed for such work. M&E Officer, Accountant, driver and community health assistants were additional positions that dedicated the most time ranging between 2% and 3.5%. Planning for HPV vaccination in Zambia thus needs to prioritise these critical positions in terms or resource allocation and support.

Demonstration project data for Gavi supported countries showed that social mobilisation and service delivery were the largest cost drivers, but this did not take into account economic costs.(16) However, this is not usually the case beyond demonstration projects when it comes to actual implementation as social mobilisation was usually heavily funded for demonstration projects but not as well funded during the actual national implementation, as have been the case in Zambia. In fact when funds are limited, activities like social mobilisation become the likely victims of reduced allocation of funds. Monitoring and evaluation is another area that is often under-funded during national rollout when funds are more limited.

One of the main drivers of cost for HPV vaccination in Africa is the cost of the vaccine itself. However, many Gavi supported countries do not bear the entire vaccine cost. Gavi, has negotiated vaccine prices with manufacturers and accessed the bivalent vaccine at US$4.50 and the quadrivalent vaccine at US$4.60 per dose.(16) In Zambia, the government only put up US$0.55 per dose as their co-finance contribution to the Gavi support. Without Gavi support, vaccine costs become a major cost driver and barrier to HPV vaccination programmes in Africa. Strategies thus need to be put in place in countries like Zambia to cater for how the vaccine cost will be funded once Gavi support ceases.

There are several methodological challenges and complexities associated with apportioning costs across HPV Vaccination delivery platforms [24, 25] which form part of the limitations of this study. Data availability and quality is one such challenge as obtaining accurate and detailed cost data can be challenging, particularly in low-resource settings where financial and administrative systems may be less developed. Incomplete or missing data can lead to inaccurate cost estimates [25, 26]. In this study, for example, supporting documents for financial data provided were not always available there was also some likelihood of recall bias given time from activity to this data collection. Secondly, allocating costs for shared resources, such as personnel, facilities, and equipment, can be complex. Different methods, such as direct allocation or step-down allocation, may produce varying results, and the choice of method can significantly impact cost estimates [24, 26]. This study settled on using EPIC data for most of the economic costs, other than human resources.

Thirdly, The costs of delivering the HPV vaccine may vary depending on factors such as geography, population density, and infrastructure. This variability can make it challenging to generalize cost estimates across different delivery platforms and settings. Lastly, the costs of HPV vaccination programs can change over time as the program scales up, achieves economies of scale, or faces changes in vaccine prices as well as fluctuations in exchange rates. Accounting for these changes in cost estimates is crucial for accurately assessing the cost-effectiveness of the program [28, 29].

The problem of cost allocation across delivery platforms if further compounded by having multiple sources of funds, including government monthly grants and special funding from donors such as Gavi, and Unite Nations agencies which may not be earmarked for any one particular delivery platform.

## Conclusion

The cost of HPV vaccination in Zambia was lowest for the school delivery platform and highest for the health facility delivery platform. Community outreach costs were in between the two though a lot lower than health facility costs. The economic cost of HPV vaccination was more than three times the financial cost across all delivery models. The main financial cost drivers were microplanning, supplies and service delivery/outreach. When we consider economic costs, human resources, building overhead and vehicles were the top cost drivers. Nurses, environmental health technician and community-based volunteers were the most involved in HPV vaccination. Future planning in Zambia and other African countries with similar contexts needs to prioritise these cost drivers as well as possibly find strategies to minimise some costs. Although not a challenge now due to Gavi support, vaccine costs are a major threat to sustainability of immunisation programmes in Africa in the long run and countries must find strategies to mitigate against this.

## Figures and Tables

**Table 1:** Micro-costing for HPV vaccination in Zambia

Financial costs		
Cost category	Resource Input	Notes
1. Microplanning	Per diem, transport allowances, other allowances, fuel, venue hire, lodging, refreshments and meals, conference supplies, equipment hire, printing health cards, registers, tally sheets, distribution costs	Microplanning costs were gathered at for national and district levels
2. Training	Facilitators, resource person costs, trainers, participant per diems, support staff per diems, transport allowances, other allowances, venue hire, lodging, refreshments, conference supplies, equipment hire, printing of health cards, tally sheets and registers, other materials	Training at national and district levels as well as health facility level
3. Service delivery	Per diem to vaccinators, project management and support staff; fuel and lubricants, Vehicle maintenance and/or repair costs, vehicle hire, other vehicle costs, waste management, M&E, carriers, ice packs, tents and materials, banners, other costs, laboratory supplies	Service delivery applied to the vaccination week and to all delivery models (school, health facility, community outreach)
4. Vaccines and supplies	Doses received 2019 and 2020; Doses used in 2019 and 2020	Vaccine costs related to co-financing obligation at USD 0.55 per dose as Gavi bore the major part of the cost
5. Cold chain specifically purchased for HPV vaccine	Purchase price, maintenance, electricity, fuel	Cold chain specifically for HPV vaccine in 2019 and 2020
6. Waste management	Safety boxes, plastic bags, gloves, other protective gear, sterilisation chemicals, other materials	Waste management supplies specifically bought for HPV vaccine
Economic costs		
Cost category	Cost elements	
7. Other Cold chain used for HPV vaccine	Purchase price, electricity, proportion used for HPV vaccine, maintenance, fuel	All economic costs obtained from EPIC study and were outlined as cost per facility. Thus the costs obtained were multiplied by number of facilities and then apportioned to HPV as 50% in that on average health facilities reported using half the child health week for HPV vaccination activities.
8. Other equipment	Total and proportion used for HPV vaccine	
9. Building overhead	Total and proportion used for HPV vaccine	
10. Vehicles	Total and proportion used for HPV vaccine	Human resource data collection focused on time spent on the activities listed by each health cadre and apportioning costs to this time based on the salary scale of the cadre
11. Vehicle maintenance	Total and proportion used for HPV vaccine	
12. Human Resources	Receiving training, conducting training, preparation and planning, Child Health Week activities, Mop-up activities after child health week, supportive supervision, data collection and reporting, community mobilisation, surveillance relating to HPV. Cadres included District Director of Health, Medical Doctor, Medical Licenciate, Health Information Officer, MCH coordinator, Health promotion officer, clinical officer, planner, M&E officer, Accountant, Logistician, Environmental Health Technologist, Registered Nurse/Midwife, Driver, Community health assistant, Community Based Volunteers, Nursing officer, Adolescent Health focal point person, Nutritionist, Nursing Officer MCH, HMIS officer, Others	

**Table 2:** HPV Delivery models in Zambia

Delivery model	Number of sites	Percentage
Health facilities	327	15.8%
Community outreach sites	641	30.9%
Schools	1108	53.3%
**Total**	**2076**	**100.0%**

**Table 3:** HPV Vaccine Coverage 2020

COVERAGE	Sum	Overall % (first and second dose)
**First Dose**	**25,308**	
*Schools*	21,874	93.0%
*Community Outreach Sites*	947	6.0%
*Health Facilities*	76	1.0%
**Second Dose**	**21,787**	
*Schools*	19,390	**Dose 2**
*Community Outreach Sites*	1,706	0.46
*Health Facilities*	364	

**Table 4:** Costs attributable to HPV Vaccination in Zambia, 2020

B. COSTS	Sum	Average/District	Dose 2 Costs (46%)
**Planning and Training**			
*Microplanning*	47,595	*5,949*	22,018
*Training*	23,266	2,908	10,763
Orientation			
Orientation/Training	8,424	1,053	3,897
Social Mob and Service Delivery			
*Social Mobilisation*	5,535	692	2,561
*Service delivery/Outreach*	48,162	6,020	22,281
*Supervision & M&E*	4,602	575	2,129
*communication*	436	54	202
Vaccines and Supplies			
*Supplies (safety boxes, needles, syringes etc)*	77,909	9,739	36,043
*Vaccines Co-finance*	25,902	3,238	11,983
*Health Cards + Registers + Tally Sheets + Other materials*	3,224	403	1,491
*Distribution*	956	119	442
*Other costs*	982	123	454
Model specific costs			-
*School Outreach*	9,545	1,193	4,416
*Health Facility*	4,308	539	1,993
*Community Outreach*	5,355	669	2,477
Other costs apportioned (economic)			-
*Cold chain maintenance and utilities*	19,838	2,480	9,177
*Sterilisation and Waste management*	37,304		
*Cold chain equipment*	60,332	7,541	27,911
*Cold Chain Energy Costs*	32,373		
*Other equipment*	86,001	10,750	39,786
*Building overhead*	121,317	15,165	56,124
*Vehicles*	105,458	13,182	48,787
*vehicle maintenance*	19,293	2,412	8,925
*Human Resources*	274,220	34,277.5	126,860
TOTAL (USD) including HR	**1,022,337**	**119,082**	**472,957**

**Table 5:** HPV vaccinations cost per dose and perf fully immunised child in Zambia

Costs apportioned to delivery model	Cost (USD)	Doses administered	Cost per dose (USD)	Cost per FIC (2 doses, USD)
**Economic Cost**				
TOTAL	1,022,337 (100%)	47,095	23.0	46.0
Health facilities	161,033 (15.8% of total)	440	365.2	730.4
Outreach	315,664 (30.9% of total)	2,652	118.8	237.7
Schools	545,640 (53.4% of total)	41,264	13.2	26.4
**Financial Cost**				
TOTAL	266,202 (100%)	47,095	6.0	11.9
Health facilities	41,931 (15.8% of total)	440	94.7	189.4
Outreach	82,194 (30.9% of total)	2,652	30.8	61.6
Schools	142,077 (53.4% of total)	41,264	3.4	6.8

**Table 6:** Cost of HPV vaccine per dose across some African countries

Year (reference)	Country	Economic cost per dose US$	Financial cost per dose US$
2021(14)	Senegal	12.24	7.75
2018(15)	Zimbabwe	45.00	19.76
2012(16)	Tanzania	10.62	5.68
2012(18)	Tanzania (Mwanza)	3.09	1.73
2019(20)	Mozambique	24	-

## Data Availability

Data is available on request from the corresponding author.

## Abbreviations

CBV: Community-based volunteer
CHW: Child Health Week
EHT: Environmental Health Technologist
EPI: Expanded Programme on Immunisation
EPIC: Expanded Programme for Immunisation Costing and Financing Project
FCE: Full Country Evaluation
FIC: Fully Immunised Child
HPV: Human Papilloma Virus
M&E: Monitoring and Evaluation
MOH: Ministry of Health
ZITAG: Zambia Immunisation Technical Advisory Group
